# Moxa Wool in Different Purities and Different Growing Years Measured by Terahertz Spectroscopy

**DOI:** 10.34133/2022/9815143

**Published:** 2022-05-31

**Authors:** Yongni Shao, Di Zhu, Yutian Wang, Zhi Zhu, Wenchao Tang, Zhengan Tian, Yan Peng, Yiming Zhu

**Affiliations:** ^1^Terahertz Technology Innovation Research Institute, Terahertz Spectrum and Imaging Technology Cooperative Innovation Center, Shanghai Key Lab of Modern Optical System, University of Shanghai for Science and Technology, Shanghai 20009, China; ^2^Shanghai Institute of Intelligent Science and Technology, Tongji University, Shanghai 200092, China; ^3^School of Acupuncture-Moxibustion and Tuina, Shanghai University of Traditional Chinese Medicine, China; ^4^Shanghai International Travel Healthcare Center, Shanghai Customs District P.R. 200335, China

## Abstract

Moxa wool is a traditional Chinese herbal medicine, which can warm channels to dispel coldness. At present, there is no unified index to evaluate the purity and growing years of moxa wool in the market. Terpineol is one of the effective substances in the volatile oil of moxa wool. Here, we characterize the purity and growing years of moxa wool by studying terpineol. Gas chromatography-mass spectrometry (GC-MS) and high-performance liquid chromatography (HPLC) are the methods for monitoring terpineol at present, all of which have defects of complicated procedures. We established linear fitting to distinguish the different purities of moxa wool through the intensities (areas) of terpineol, the characteristic peaks, and the consequence presented; the coefficient of determination (*R*^2^) was higher than 0.90. Furthermore, based on the characteristic peak position of standard terpineol, the correlation model with the purity and growing year of moxa wool was set up, thereby differentiating the quality of moxa wool. We have built the partial least squares (PLS) model of the growing years of moxa wool with high accuracy, and the determination coefficient is greater than 0.98. In addition, we compare the quantitative accuracy of Raman spectroscopy with terahertz technology. Finally, a new method of terahertz spectroscopy to evaluate quality of moxa wool was found. It provides a new idea for the identification of inferior moxa wool in the market and a new method for identifying the quality of moxa wool in traditional Chinese medicine.

## 1. Introduction

Wormwood has the effects of relieving dampness, blood stasis, and swelling, which have been widely applied to traditional Chinese medicine [1, 2]. Among them, moxa wool is an important wormwood product, obtained by repeatedly processing wormwood to remove powder residue and its purity (wormwood-to-moxa wool ratios) and growing year determine its quality [3]. Generally, easily volatile substances in moxa wool decrease with the increase of purity and growing years of moxa wool. The difficult volatile substances in moxa wool are the opposite. Generally, difficult volatile matters have expectorant, antiasthmatic, and bacteriostatic curative effects. The price of moxa wool increases with the increase of curative effect. The prices of one-year growth period, two-year growth period, three-year growth period of moxa wool, and so on increased by 20 yuan in turn. At present, there is no unified evaluation index in the market to evaluate the purity and growing years of moxa wool. Terpineol is one of the effective substances in the volatile oil of moxa wool, which presents microbicide, anticancer, and other biological actions [4]. The determination of terpineol in moxa wool is of great significance to distinguish moxa wool quality. Thus, we studied the purities and growing years of moxa wool through the change of terpineol.

Most current methods for detecting terpineol are usually chemical, such as GC-MS and HPLC [5, 6]. The results of GC-MS analysis are accurate and reliable, but the pretreatment and analysis steps are complicated [7]. HPLC is on the basis of disconnected substances, which depends on the difference of adsorption characteristics and protein molecular size and usually requires a long detection time and high sample preparation cost [8].

Besides, the aforementioned terpineol celluloid-tested approaches, an increasing number of approaches of spectrum detection are applied to biological detection, consisting of infrared spectroscopy and Raman spectroscopy [9]. Infrared spectrum, on account of different substances, has different absorption intensities and wavelengths; their reflected absorption peaks and intensities in infrared spectrum are different from each other [10, 11]. J. Li et al. quantitatively analyzed volatile lignans by infrared spectroscopy [12]. Moni et al. use FTIR spectral analyzer to certificate the existence of alkaloids and tannins [13]. These consequences displayed that infrared spectroscopy technology supplied a valid and practicable method for the test of botanical physical message. Raman spectrum can reflect the difference of chemical composition of samples at molecular level [14]. Different detection method pairs are shown in Table 1. Yoon et al. studied Raman spectroscopy which is a new method to detect terpineol as an effective component in new green tea essential oil nanoemulsion [15]. These researches present that it is feasible to analyze moxa wool by terpineol characteristic absorption peak spectrum.

Happened along with development wake of modern ultrafast optics, THz spectroscopy is becoming gradually full grown. Terahertz wave is an electromagnetic wave, which owns a frequency scope between 0.10 and 10.0 THz and the properties of infrared wave and microwave [18]. A great deal of biological samples has obvious THz characteristic absorption peak, and THz technology has added preponderance in the vibration and rotation level of macromolecules [19]. THz spectroscopy is in the extreme impressive to polar crowds. Inverse to Raman spectroscopy, which chiefly represents oscillation inside the molecule, the spectrum message in THz domain is ample at frail inside the molecule mutual and skeleton oscillation of macromolecules, which is straightway relevant to molecular configuration [20, 21]. For this reason, THz technology is more and more used to characterization and dissect of configuration.

The near-infrared spectrum bands overlap seriously, and the number of peaks in near-infrared spectrum is generally relatively large, and the same band is often overlapped by multiple groups, and the -CH_3_ and -OH groups of terpineol overlap, which leads to the near-infrared spectrum of terpineol with severe overlapping peaks and wide peaks. Raman scattering area has great influence on the analysis, and the overlapping of different vibration peaks and Raman scattering intensity is easily influenced by optical parameters and other factors. The terahertz band lies between the millimeter wave band and the far-infrared band. From the energy point of view, the frequencies corresponding to the weak interaction between molecules, the skeleton vibration of macromolecules, and the low-frequency vibration absorption of crystal lattice are just within the terahertz band. Therefore, we propose a new method to use terahertz technology to identify the quality of moxa wool by terpineol.

Recently, THz technology has been applied to study Chinese herbal medicines, including Yin et al. who used terahertz spectroscopy to study the biological molecular characteristics of baicalein, quercetin, and other common flavonoids in 0.2-2.5 THz band [22]. Yan et al. simulated the terahertz spectroscopy of glycyrrhizic acid by quantum chemical calculation method, and its absorption characteristic peaks matched with molecular vibration mode [23]. These studies show that the vibration modes of macromolecular compounds of these Chinese herbal medicines are matched with terahertz peaks. Kou et al. used THz spectroscopy to analyze the ginsenosides in Panax quinquefolium to differentiate herbal medicines [24]. The research shows that THz spectroscopy can be applied to test Chinese herbal medicines. At present, there is no literature report on terahertz spectroscopy used in terpineol detecting research.

Terpineol is an isomer, terahertz wave is confoundedly impressible to molecular structure, and the weak difference of isomers in terahertz wave absorption spectrum can be obviously different. We have obvious advantages in detecting isomer terpineol by terahertz spectroscopy. In addition, to further enhance prediction accuracy of the model of moxa wool, we improved sample preparation method. COC (cycloolefin copolymer) has extremely low transmission loss compared with polyethylene (PE) spectral information in terahertz band [25] and has the advantages of high transparency and low water absorption [26]. To solve these technical bottlenecks of terahertz measurement, we mixed cycloolefin copolymer (COC) instead of polyethylene (PE) with moxa sample and tableted it. Further, in the process of detecting volatile liquid terpineol, we tried various methods, dropping terpineol on filter paper and silicon chip and weighing paper, finally reducing the volatility of terpineol in the detection process by making a sample cell and successfully detecting the terahertz spectrum of terpineol. It provides the possibility for the next step of modeling and improving the modeling accuracy.

During the research of this paper, we put forward a fast-nondestructive testing method of moxa wool based on terahertz spectrum. Firstly, the vibration absorption spectrum of terpineol molecule with high content in moxa wool was simulated by DFT, and the characteristic peak position of terpineol in terahertz band was identified. Then, Fourier transform infrared spectroscopy (FTIR) is used to collect THz spectra of moxa wool in different growing years and different purities. Find the characteristic peak position of terpineol corresponding to moxa wool. Therefore, we established the purity model of moxa wool with peak heights and peak areas, respectively. Then, we also use the PLS model, establishing the relevant model of the purities and growing years of the moxa wool, and compare the prediction accuracy of the two models. Moreover, we have done Raman spectroscopy experiments to detect moxa wool in different growing years and different purities and try to compare the quantitative accuracy of the two technologies. The model based on the characteristic peaks of terpineol can accurately distinguish the growth years and purity of moxa wool. We can solve the problem of shoddy moxa wool in the current market, moreover identifying the best quality of moxa wool for traditional Chinese medicine.

## 2. Materials and Methods

### 2.1. Chemical Reagent

The molecular formula of terpineol is C_10_H_18_O, and its molecular weight is 154.24, which is insoluble in water and purchased from Aladdin. The reagent purity is more than 98%, colorless viscous liquid; CAS number is 8000-41-7. All the tested moxa wool is extracted from the moxa sticks (Nanyang Hanyi Moxa Co., Ltd., Henan, China) 18 mm in diameter, 200 mm in length, and 21-22 g in mass. The wormwood-to-moxa ratio of the moxa sticks with different storage years (five, eight, and ten years) which are numbered as “2017,” “2014,” and “2012,” respectively, was 15 : 1, and the storage years of the moxa sticks in different wormwood-to-moxa ratios (10 : 1, 15 : 1, and 30 : 1) were 8 years.

### 2.2. Density Functional Theory

Density universal function theory (DFT) is a common method used to analyze molecular vibration and rotation [27]. The theoretical origin of terpineol peak position was explained by DFT combined with terpineol molecular structure, and the experimental data of terpineol was further explained. Because of its high precision and low computational complexity, the DFT plays a significant role in the analysis of molecular structure [28]. Using Gauss -09 package (revised version D.01, American Gauss Company) together with B3LYP hybridization function and 6-31G basis set, we calculated the absorption peak position of terpineol at THz frequency [29].

### 2.3. Sample Preparation and Spectral Collection

Firstly, the moxa wool samples were ground, and the steel balls with diameter of 2 mm and moxa wool were put into a grinding tube, and the moxa wool was ground for 190 s by a grinder. (The parameter of the grinder machine is composed of frequency and grinding time, which are set to 70 Hz and 190 s.) Secondly, the ground moxa wool mixed with COC powder is pressed into tablets (20 mg moxa wool mixed with 40 mg COC), the quality of each tablet is controlled at 60 mg, and the experimental quality loss is controlled within 1%. When tableting, press moxa wool powder in a die with a stress of 4 tons for two minutes to take shape, a thin slice with a thickness of 1 mm.

For the same batch of different growing years of the moxa wool samples, three varieties with different growing years of 2012, 2014, and 2017 were selected, and each variety suppressed 15 samples, with a total of 45 samples from one batch. For different purities of moxa wool, which were 10 : 1, 15 : 1, and 30 : 1, each variety will also prepare 15 samples. We totally prepared three batches of moxa wool samples, including the different growing years and purities of moxa wool. Two of these three batches are used to build the moxa wool model, and another batch of moxa wool with different growing years and different purities is bought from the market to verify the accuracy of the model. Terahertz signal is easily assimilated by water vapor, so it is necessary to dry the internal part of the experimental apparatus before performing an experiment. Guarantee that the whole terahertz signal survey section is in an environment with moisture content less than 3% [30]. Fourier transform infrared spectroscopy (FTIR) was applied to take a measurement to the absorption spectrum. Water-cooled mercury lamp is the light source. FTIR has a signal-to-noise ratio of surpassing 10000 : 1 and a frequency domain of 0.9-20.0 THz. The resolution of the spectrometer is 4 cm^−1^, and every spectroscopy is the average of 64 sample sweeps applied to a sweep velocity of 5 kHz in the view of 64 backdrop sweeps. In the process of collecting spectral data, the apparatus is full of nitrogen to eliminate the influence of moisture on terahertz spectral collection. FTIR has excellent scanning accuracy, and the jitter rate is under 3% in the measurement process. Before measuring the spectrum, it is necessary to scan the background and eliminate the background and record the scanning results by using the spectrum software OPUS. In the process of gathering moxa wool spectra, 3 diver places were gathered for each tablet, 5 times for each point, and then, the average value was taken.

In addition, the advantages and disadvantages of terahertz spectra are compared by Raman spectroscopy, which is consistent with the sample preparation method of terahertz spectroscopy. For moxa wool samples from different growing years (2012, 2014, and 2017), three batches of samples were prepared. For the different purities of moxa wool samples (10 : 1, 15 : 1, and 30 : 1), there are 45 samples in one batch (15 samples in each proportion) and 3 batches in total. Using laser confocal micro-Raman spectroscopy, the optical effectiveness is raised above 30.0%, the spectrum resolving power exceeded 0.40 wave number, and the aclinic resolving power is super than 1 micron. We employ 532.0 nm optical maser as Raman optical maser and 2.50% strainer. The time of acq and RTD are 5.0 s and 1.0 s. As to every last sample slice, we gather 3 different situations, and every last slice was gathered 5 times and thereafter equalized.

### 2.4. Partial Least Squares Algorithm

PLS, a dimension reduction technique, is used to maximize the covariance between the prediction (independent) matrix *x* and the prediction (correlation) matrix *y* on each component of the dimension reduction space. PLS improves the original least squares algorithm and cannot use all variable data in calculation. The principal component extraction algorithm is applied to drop dimension of the original data, so as to extract metadata to the maximum extent and extract the main information from the residual error.

## 3. Results

### 3.1. Molecular Simulation of Terpineol

The spectral information of terpineol molecules in terahertz band is calculated and simulated by Gaussian-09 software and density functional theory [31–33]. In addition, the origin of characteristic absorption peaks of the terpineol was simulated through the assistance of optical window [34].

**Figure 1:**
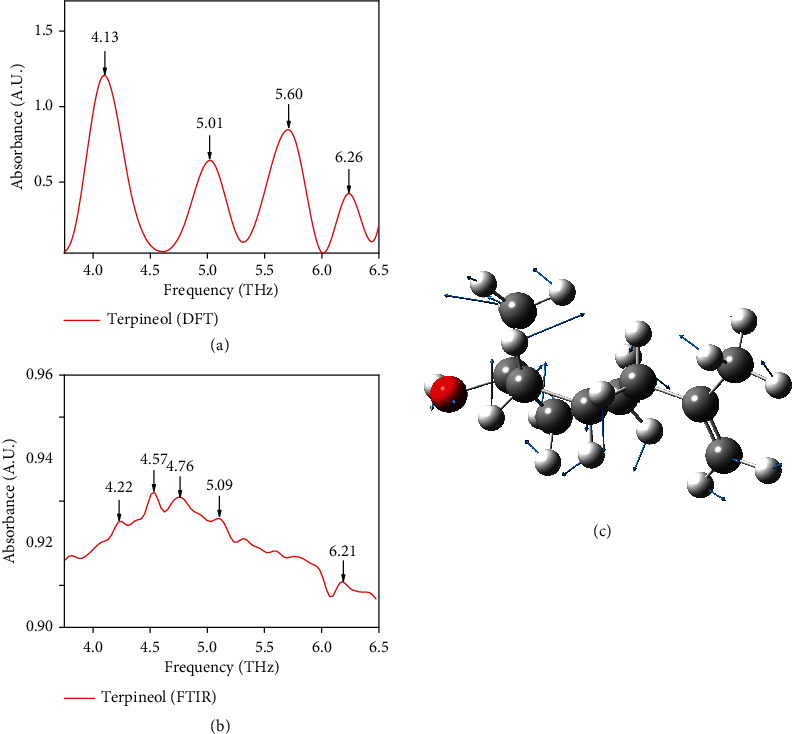
(a) THz absorption spectroscopy of terpineol on the basis of DFT; (b) THz absorption spectroscopy of terpineol on the basis of experiment; typical vibration modes at (c) 4.17 THz; black, grey, and red atoms represent carbon, hydrogen, and oxygen atoms, respectively. The blue arrows indicate the vibration direction of atoms, and the length of the arrow indicates the vibration amplitude of atoms.

The absorption spectrum of terpineol received by theoretical calculation is shown in Figure 1(a), and the absorption spectrum of terpineol received by molecular simulation is shown in Figure 1(b). In the experimental results of terpineol, terpineol is mixed according to the isomer ratio of 1 : 1 : 1, including *α*-terpineol, *β*-terpineol, and *γ*-terpineol. As shown in Figure 1(a), through molecule simulating, we can find that there are four characteristic peaks of terpineol, which are situated at 4.13, 5.01, 5.60, and 6.26 THz. In the results of the experiment from Figure 1(b), we can find five characteristic peaks, which are situated at 4.22, 4.57, 4.76, 5.09, and 6.21 THz. By the viewable window of Gaussian, we can find that this oscillation at 4.13 THz is causal for folding vibration of molecular as shown in Figure 1(c). We compare the simulational consequences of terpineol with the tested consequences to further analyze the standard terpineol; we discovered that the absorption peaks at 4.13 THz and 5.01 THz calculated theoretically by the standard terpineol are equivalent to the absorption peaks at 4.22 THz and 5.09 THz detected experimentally, respectively. The absorption peaks at 4.13 THz and 5.01 THz of simulated spectral are weakly red shift contrast to the measured spectral. The reason of red-shifted hydrogen bond is mainly due to elongation and contraction effect [35]. The combination of covalent bonds and electronegative atoms is the main combination mode of hydrogen atoms. We observe that the absorption peak position of 5.60 THz calculated by theory is not reflected in the spectrum detected by experiment, which may be connected to temperature through analysis [36]. In molecular systems, hydrogen bonds are easily impinged by temperature, which further leads to molecular vibration frequency become different, thus leading to the frequency shift of peak position [37]. In addition, the absorption peaks at 4.57 THz and 4.76 THz detected in the experiment did not present in simulated consequences. The standard terpineol detected in the experiment is a multimolecular system, which includes not only the atomic vibration of single molecule but also the association between molecules [38]. The interaction between molecules in a multimolecular system will lead to the absorption of characteristic peaks of terahertz waves [39]. Therefore, the theoretical model verifies that terpineol can be detected in terahertz spectrum range, which lays a foundation for the follow-up research of moxa wool.

### 3.2. Study on Different Purities of Moxa Wool

**Figure 2:**
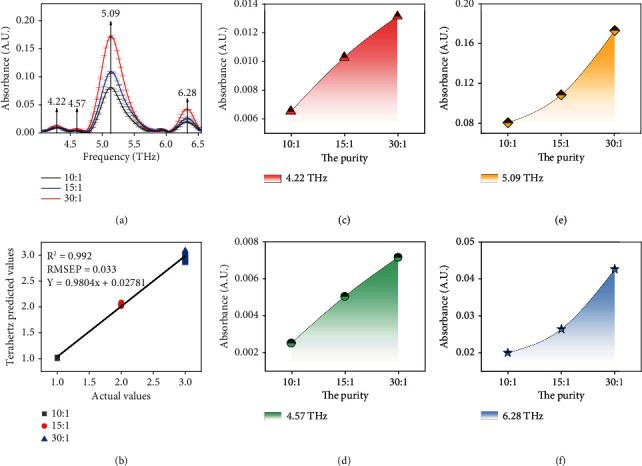
(a) THz absorption spectral on the basis of the different purities of moxa wool. Vertical arrows indicate absorption peaks of moxa wool. Error bars have been labeled on each data. (b) PLS prediction model of moxa wool in different purities based on terahertz absorption spectrum. Characteristic absorption peak intensities on different purities of moxa wool at (c) 4.22 THz, (d) 4.57 THz, (e) 5.09 THz, and (f) 6.28 THz.

Afterwards, deleting baseline and delineating by thickness of samples, terahertz spectroscopy of moxa wool in different purity is displayed in Figure 2. From Figure 2(a), we know that moxa wool has four characteristic absorption peaks in the 4-6.5 THz band, which are 4.22, 4.57, 5.09, and 6.28 THz, respectively.

We compared the absorption peaks of moxa wool with terpineol. The absorption peaks of moxa wool at 4.22, 4.57, 5.09, and 6.28 THz correspond to that of terpineol at 4.22, 4.57, 5.09, and 6.21 THz. Among them, compared with the standard terpineol, the corresponding absorption peak at 6.28 THz on moxa wool is offset by 0.07 THz. Through analysis, when the absorption peaks of some hydrogen-containing groups overlap with those of some groups, it is possible to deuterate the hydrogen of the functional groups, so that the absorption peaks shift to low wave number [40].

Different states of the same molecule and their interactions between molecules are different, which leads to different detected spectra [41]. Generally, the wave number of the spectral band measured in gaseous state is the highest, and the rotating fine structure of the vibrating spectral band can be observed. At this time, the moxa wool is in solid state, and the wave number will be lower than that of terpineol measured in liquid state. Therefore, the frequency shift of moxa wool peaks may be caused by many factors.

To go a step further, analyze the cause for variation tendency of the intensity of absorption peaks with different purities of moxa wool, as shown in Figures 2(c)–2(f); we can clearly find that with the increase of the purities of moxa wool, the intensity of absorption peaks shows an increasing trend. For the different purities of moxa wool, with the increase of the purity of moxa wool, the relative content of terpineol increases regularly. Through the analysis, the relative content of terpineol increases with the increase of moxa wool purities. Hence, we can consider that the change of relative terpineol content leads to the change of absorption peak intensity of different purities of moxa wool.

In addition, according to the correspondence between terpineol and the peak positions of moxa wool, we selected three characteristic peak positions of moxa wool as 4.22, 5.09, and 6.28 for further modeling and analysis.

#### 3.2.1. Linear Fitting of Moxa Wool with Different Purities

**Figure 3:**
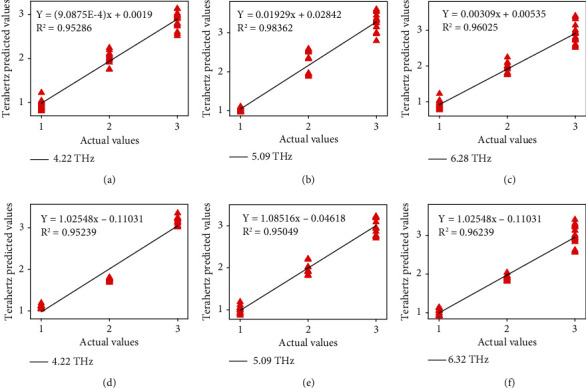
Linear fitting prediction consequences of characteristic absorption peak intensities on different purities of moxa wool at (a) 4.22 THz, (b) 5.09 THz, and (c) 6.28 THz. Linear fitting prediction results of characteristic absorption peak areas on different purities of moxa wool at (d) 4.22 THz, (e) 5.09 THz, and (f) 6.28 THz.

Linear fitting is used to model and analyze moxa wool with different purities. Moxa wool prediction model is established according to set calibration and prediction set of 2 : 1. A total of 60 samples (20 samples for each variety) were applied to calibrate, and 30 samples (10 samples for each variety) were applied to predict, and the purities of 10 : 1, 15 : 1 and 30 : 1 are numbered as “1,” “2,” and “3,” respectively. We use the characteristic peak intensity and characteristic peak area as two parameters to linear fitting the peak height (area). Three absorption peaks of 4.22, 5.09, and 6.28 THz are regarded as characteristic absorption peak intensities, and three integral regions of 4.10-4.48 THz, 4.76-5.89 THz, and 6.09-6.56 THz are taken as peak areas (such as Table 2, linear fitting results of different purity moxa wool, and Figure 3, linear fitting prediction results of different purity moxa wool).

As shown in Table 2, *x*, *Y* and *R*^2^ are the purity of the sample, the absorption peak intensity (or area) of the sample, and the determination coefficient, respectively. From Figure 3, for the prediction of characteristic absorption peak heights, we can find that the coefficient of determination at 5.09 THz reaching 0.983 is the highest among the three characteristic absorption peaks; for the prediction of characteristic absorption peak areas, integral region of 6.09-6.56 THz is the highest, in which the coefficient of determination can reach 0.962. The determination coefficient *R*^2^ in the modeling results of different purity moxa wool are greater than 0.95, which indicates that terahertz technology has the ability to quantitatively detect the purity of moxa wool.

#### 3.2.2. Partial Least Squares Modeling of Moxa Wool with Different Purities Based on Terahertz Spectrum

Firstly, we analyze and obtain the terahertz spectrum of moxa wool, remove the thickness of the obtained spectrum during processing, and then smooth and remove the baseline, which can correct the baseline fluctuation and enhance the signal-to-noise ratio (SNR).

Moreover, PLS regression prediction model was established by us to predict different purities of moxa wool, in order to enhance the precision of the model. The characteristic peak intensities of the same three characteristic peaks at 4.22, 5.09, and 6.28 THz are used as the input of PLS model; outputs correspond to “1,” “2,” and “3,” respectively. A total of 60 samples (20 samples for each variety) were applied to calibrate and 30 samples (10 samples for each variety) were applied to predict.

In the end, the precision of the model is assessed by the determination coefficient (*R*^2^) and root mean square (RMSE). The verification includes correction of correlation coefficient and full cross-validation of RMSE. In order to verify the correction model, the model is applied to the spectral data of the prediction set to obtain the prediction root mean square (RMSEP) and determination coefficient (*R*^2^). The purity of PLS modeling and prediction samples is consistent with that of linear model. It can be found from Figure 2(b) that the *R*^2^ of the prediction model with different purities of moxa wool all oversteps 0.990, and the RMSE is under 0.05. In contrast to the linear fitting outcomes, the outcomes show that the *R*^2^ of PLS modeling prediction goes beyond 0.990, which oversteps that of linear fitting 0.950.

### 3.3. Study on Different Growing Years of Moxa Wool

**Figure 4:**
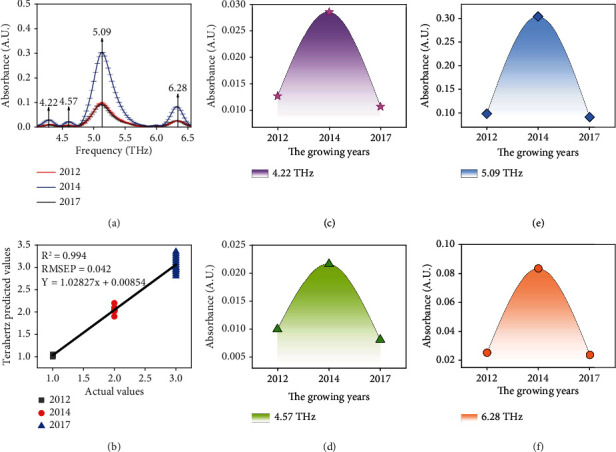
(a) THz absorption spectral on basis of different growing years of moxa wool. Vertical arrows indicate absorption peaks of moxa wool. Error bars have been labeled on each data. (b) PLS prediction model of moxa wool in different growing years based on terahertz absorption spectrum. Changes of characteristic absorption peak intensities on different growing years of moxa wool at (c) 4.22 THz, (d) 4.57 THz, (e) 5.09 THz, and (f) 6.28 THz.

We have studied different growing purities of moxa wool and want to further study different growing years of moxa wool (7-year growth period 2014 moxa wool). As shown in Figure 4(a), there are four characteristic absorption peaks at 4.22, 4.57, 5.09, and 6.28 THz of different growing years of moxa wool. The characteristic absorption peaks of moxa wool in different growing years are the same as those of moxa wool in different purities.

We further analyze the reason for variation tendency of the intensity of absorption peak different growing years of moxa wool, as shown in Figures 4(c)–4(f); we can clearly find that with the increase of the years of moxa wool, the absorption peak intensity of moxa wool increased at first and then decreased. Through the analysis, the relative content of terpineol increases first and then decreases with the increase of moxa wool years. And in 2014, the terpineol content of moxa wool was the highest. Therefore, the change of terpineol relative content leads to the change of absorption peak intensities of moxa wool in different purities.

In addition, according to the correspondence between terpineol and the peak positions of moxa wool, we selected three characteristic peak positions of moxa wool as 4.22, 5.09, and 6.28 THz for further modeling analysis.

### 3.4. Partial Least Squares Modeling of Moxa Wool with Different Growing Years Based on Terahertz Spectrum

We set up a partial least squares regression prediction model for different growing years to predict. The characteristic peak intensities of the three characteristic peaks at 4.22, 5.09, and 6.28 THz are regarded as the input of PLS model; outputs correspond to “1,” “2,” and “3,” respectively. It is constructed according to the ratio of calibrated set to predicted set of 2: 1. A total of 60 samples were applied to calibrate, and 30 samples were applied to predict. It will be obtained from Figure 4(b) that the *R*^2^ of predictable model with different growing years of moxa wool is all above 0.990, and the RMSE is less than 0.05.

### 3.5. Analysis of Raman Spectra

#### 3.5.1. Analysis of Raman Spectra in Terpineol

After using THz technology to analyze the different growing years and different purities of moxa wool through the change of terpineol, we try to use the Raman spectroscopy to analyze moxa wool in different growing years and different purities through the change of terpineol. Ultimately, the results of the two technologies are compared.

**Figure 5:**
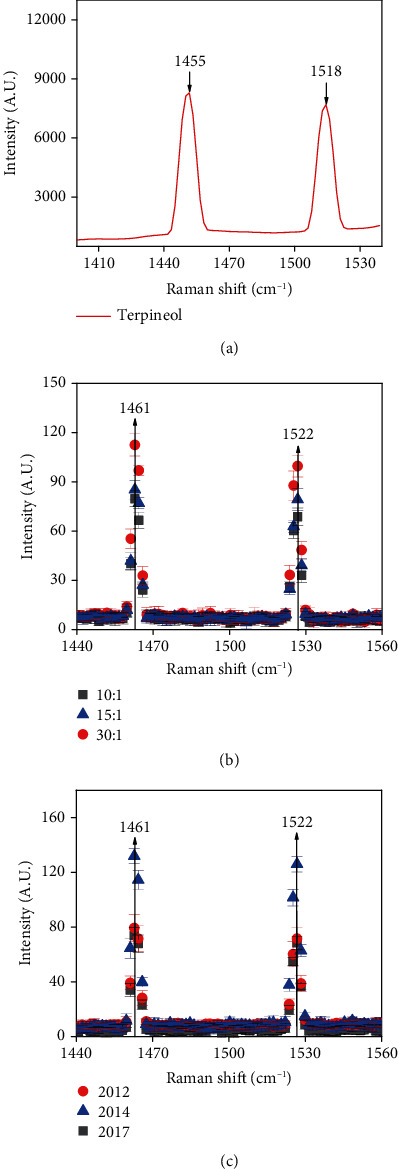
Raman spectrum curves based on the (a) terpineol. (b) Different growing purities of moxa wool. (c) Different growing years of moxa wool. Vertical arrows indicate absorption peaks of moxa wool. Error bars have been labeled on each data.

From Figure 5(a), it is the Raman spectroscopy of terpineol, which have two obvious characteristic absorption peaks in the band of 1400 cm^−1^-600 cm^−1^, which are located at 1455 cm^−1^ and 1518 cm^−1^, respectively. It is found that the characteristic absorption peak at 1455 cm^−1^ may be due to asymmetric angular vibration of CH_3_. The absorption peak at 1518 cm^−1^ may be the variable angle of CH [42].

#### 3.5.2. Analysis of Raman Spectra in Different Purities of Moxa Wool and Different Growing Years of Moxa Wool

In order to use Raman spectroscopy to analyze moxa wool in different growing purities and different growing years through the change of terpineol, the Raman spectroscopy of moxa wool was collected.

From Figures 5(b) and 5(c), it is the Raman spectra of moxa wool, which have two obvious characteristic peaks in the band of 1400 cm^−1^-600 cm^−1^, which are located at 1461 cm^−1^ and 1522 cm^−1^, respectively. Comparing the Raman spectra of Figure 5(a) in terpineol, we can find that the peak positions of the Raman spectra of moxa wool are 1455 cm^−1^ and 1518 cm^−1^, which correspond to the characteristic peaks of terpineol 1455 cm^−1^ and 1518 cm^−1^, respectively. Because the characteristic frequency of small groups bound atoms is not completely independent of other parts of the molecule, the vibration of each group is generally in a range. The vibration frequency of CH_3_ is 1455 cm^−1^-1465 cm^−1^; the vibration frequency of CH is 1515 cm^−1^-1525 cm^−1^ [43, 44]. It is explained that the characteristic absorption peaks of moxa wool at 1461 cm^−1^ and 1518 cm^−1^ correspond to the characteristic peaks position of terpineol at 1455 cm^−1^ and 1522 cm^−1^ with a frequency shift. For this reason, we can obtain that these two absorption peaks are resulted in terpineol.

For the different purities of moxa wool, from Figure 5(b), with the increase of moxa wool purities, the absorption peak intensity of moxa wool increases. Moreover, with the purities of moxa wool, the terpineol content increases regularly. We can get a conclusion that the change of characteristic absorption peak intensity of different purities moxa wool is caused by the change of terpineol content.

We further analyze the reason for variation tendency of the intensity of absorption peak different growing years of moxa wool; we can clearly find from Figure 5(c) that with the increase of moxa wool years, the absorption peak intensity of moxa wool increases at first then decrease. The truth is with the increase of moxa wool growing years, the terpineol content increases at first and then decreases; in the 2014, the terpineol content is the highest. Hence, the change of terpineol content gives rise to the increase of characteristic absorption peak intensity.

#### 3.5.3. PLS Modeling of Moxa Wool with Different Purities Based on Raman Spectroscopy

**Figure 6:**
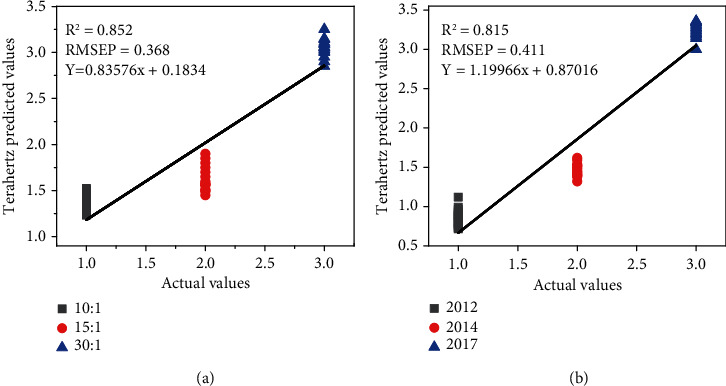
PLS prediction model of moxa wool in (a) different purities and (b) different growing years based on Raman spectrum.

We also wanted to make a contrast to the precision of these two techniques for moxa wool in different purities. The characteristic absorption peak intensities at 1461 cm^−1^ and 1522 cm^−1^ were selected to establish PLS model. A total of 60 samples were applied to calibrate, and 30 samples were applied to predict. The analysis results are shown in Figure 6(a); the *R*^2^ of the model is 0.852, which is far from the terahertz model (*R*^2^ > 0.95). Hence, THz spectroscopy has palpable superiorities in measurable analysis of moxa wool purities.

#### 3.5.4. PLS Modeling of Moxa Wool with Different Growing Years Based on Raman Spectroscopy

We choose moxa wool in different growing year corresponding to the characteristic peak intensities of terpineol at 1461 cm^−1^ and 1522 cm^−1^ to establish PLS model to compare the prediction accuracy of terahertz technology and Raman spectroscopy. A total of 60 samples were applied to calibrate, and 30 samples were applied to predict. The analysis results are shown in Figure 6(b); the *R*^2^ of the model is 0.815, which is far below from the terahertz fitting model (*R*^2^ > 0.95). Therefore, THz technology has evident preponderances in quantitative analysis of moxa wool growing years.

### 3.6. Different Growing Years and Different Purities of Moxa Wool in the Market Are Predicted, Based on the Established PLS Theoretical Model

For the sake of testing and verifying the prediction accuracy and reliability of the model of moxa wool in different purities and different growing years in Sections 3.3, 3.4, and 3.5, we bought different purities of moxa sticks in different wormwood-to-moxa ratios (10 : 1, 15 : 1, and 30 : 1) 8 years from the market, and different growing years of moxa wool which are numbered as “2017,” “2014,” and “2012,” respectively, were 15 : 1. For different purities of moxa wool samples, each variety suppressed 15 samples, with a total of 45 samples. For different growing years of moxa wool, each variety will also prepare 15 samples. Then we, respectively, collect terahertz spectra and Raman spectroscopy at specific bands. The spectral data were substituted into the PLS model for prediction, in which 5 samples were selected for each growing years (purities) for prediction. Consistent with the classification in Sections 3.3, 3.4, and 3.5, we assigned “1,” “2,” and “3” to the three growing years (three purities) in turn. After the assignment, it was determined as “1,” corresponding to the “2012,” and set the thresholds of the prediction model as 0.5 to assess the prediction precision of the moxa wool model. As shown in Table 3, it is concluded that in this experiment, the prediction accuracy in terahertz spectroscopy of different purities of moxa wool is 93.9%, and that of different growing years is 95.3%. Conversely, the Raman spectroscopy prediction accuracy is lower than THz technology. The terahertz spectroscopy model can well predict the different growth years and different concentrations of moxa wool in the market.

## 4. Discussion

In this research, we reckoned, tested, and verified the practicality of fast lossless testing of the purity and growing years of moxa wool based on terahertz spectroscopy. Firstly, linear fitting was set by using the peak height (area) of the above-mentioned three characteristic peaks with different purities of moxa wool, and the *R*^2^ in the model was outdo 0.95. Besides, we obtained a more accurate prediction model of different purity moxa wool by PLS model, and the determination coefficient *R*^2^ was greater than 0.99. Next in importance, the PLS combined the growing years of moxa wool with intensities of the terpineol characteristic peaks established, where it demonstrated an accurate model with high prediction coefficient of determination (greater than 0.98). Before we get a conclusion that the predicted accuracy of moxa wool is beyond 0.95, we try a lot of sample preparation methods, among them are grinding fineness of the sample and thickness of the sample, and we have found that the moxa wool was grinded twice (after twice grinding, the sample can be ground more fully) which is more beneficial to tableting and detection of spectral information. After grinding moxa wool, through controlling the quality of the moxa wool, we collected the best spectroscopy of moxa wool. Through improving sample preparation methods, we obtained the high prediction accuracy of moxa wool. After using the THz spectroscopy to analyze the purities and growing years of moxa wool, we repeated the above experiments by using Raman technology spectroscopy, trying to compare the quantitative accuracy for the purities and growing years of moxa wool between these two technologies. The *R*^2^ of the Raman technology spectroscopy model is above 0.80, which is far from the THz technology (*R*^2^ > 0.95). Finally, we predicted the real samples in the market and compared the prediction accuracy of terahertz technology and Raman technology. It is concluded that in this experiment, the prediction accuracy in terahertz spectroscopy of different purities is 93.9%, and that of different growing years is 95.3%. Conversely, the Raman spectroscopy prediction accuracy is lower than THz technology. Therefore, the THz spectroscopy provides a new approach to detect the purities and growing years of moxa wool and also offers a strong technical support on nondestructive monitoring of terpineol content. It provides a new idea for the identification of inferior moxa wool in the market and a new method for identifying the quality of moxa wool in traditional Chinese medicine.

## Figures and Tables

**Table 1:** Comparison of different detection methods.

Methods	Pretreatment time	Required sample size	Prediction accuracy
Gas chromatography-mass spectrometry	20 h	10 ml	<90% [16]
Raman spectroscopy	3 h	10 ml	<85% [17]
Terahertz spectroscopy	1 h	2 ml	>95%

**Table 2:** Properties of models for prediction of different purities in moxa wool using characteristic absorption intensities or areas.

Characteristic absorption peaks	Determination coefficient (*R*^2^)	Absorption intensities (*Y*)	Determination coefficient (*R*^2^)	Absorption areas (*Y*)
4.22 THz	0.97535	*Y* = 0.00956*x* − 0.00212	0.95286	*Y* = (9.0875E − 4) *x* + 0.0019
5.09 THz	0.97549	*Y* = 0.10409*x* − 0.03821	0.98362	*Y* = 0.01929*x* + 0.02842
6.28 THz	0.99055	*Y* = 0.02891*x* − 0.01303	0.96025	*Y* = 0.00309*x* + 0.00535

**Table 3:** Different purities and growing years (bought in the market) of prediction accuracy based on PLS model (threshold “0.5”).

Component	(THz) accuracy (%)	(Raman) accuracy (%)
10 : 1	90.5	75.6
15 : 1	91.2	81.2
30 : 1	100.0	77.2
2012	94.3	70.9
2014	100.0	80.0
2017	92.8	73.4
